# The impact of physical activity on progression-free and overall survival in metastatic breast cancer based on molecular subtype

**DOI:** 10.1186/s12885-024-13038-3

**Published:** 2024-10-16

**Authors:** Philipp Ziegler, Andreas D. Hartkopf, Markus Wallwiener, Lothar Häberle, Hans-Christian Kolberg, Peyman Hadji, Hans Tesch, Johannes Ettl, Diana Lüftner, Volkmar Müller, Laura L. Michel, Erik Belleville, Pauline Wimberger, Carsten Hielscher, Hanna Huebner, Sabrina Uhrig, Lena A. Wurmthaler, Carolin C. Hack, Christoph Mundhenke, Christian Kurbacher, Peter A. Fasching, Rachel Wuerstlein, Michael Untch, Wolfgang Janni, Florin-Andrei Taran, Michael P. Lux, Diethelm Wallwiener, Sara Y. Brucker, Tanja N. Fehm, Andreas Schneeweiss, Chloë Goossens

**Affiliations:** 1 Department of Gynecology and Obstetrics, Universitätsklinikum Erlangen, Friedrich-Alexander-Universität Erlangen Nürnberg, Comprehensive Cancer Center Erlangen-EMN (CCC ER-EMN), Universitätsstraße 21–23, Erlangen, 91054 Germany; 2 Department of Obstetrics and Gynecology, University of Tübingen, Tübingen, Germany; 3 Department of Gynecology, Halle University Hospital, Halle, Germany; 4 Biostatistics Unit, Department of Gynecology and Obstetrics, Universitätsklinikum Erlangen, Erlangen, Germany; 5 Department of Gynecology and Obstetrics, Marienhospital Bottrop, Bottrop, Germany; 6 Frankfurt Center for Bone Health, Frankfurt Am Main, Germany; 7 Oncology Practice, Bethanien Hospital, Frankfurt Am Main, Germany; 8 Department of Obstetrics and Gynecology, Klinikum Rechts Der Isar, Technical University of Munich, Munich, Germany; 9 Cancer Center Kempten/ Allgäu (CCKA), Klinikum Kempten, Kempten, Germany; 10 Immanuel Hospital Märkische Schweiz & Immanuel Campus Rüdersdorf, Medical University of Brandenburg Theodor-Fontane, Rüdersdorf Bei Berlin, Germany; 11 Department of Gynecology, Hamburg-Eppendorf University Medical Center, Hamburg, Germany; 12 National Center for Tumor Diseases, German Cancer Research Center (DKFZ), Heidelberg University Hospital, Heidelberg, Germany; 13 ClinSol GmbH & Co KG, Würzburg, Germany; 14 National Center for Tumor Diseases Dresden and Department of Gynecology and Obstetrics, University Hospital Dresden, TU Dresden, Dresden, Germany; 15 g.SUND Gynäkologie-Onkologisches Zentrum, Stralsund, Germany; 16 Department of Gynecology and Obstetrics, Klinik Hohe Warte, Bayreuth, Germany; 17 Department of Gynecology I (Gynecologic Oncology), Gynecologic Center Bonn-Friedensplatz, Bonn, Germany; 18 Department of Gynecology and Obstetrics, Breast Center and CCC Munich, University Hospital LMU Munich, Munich, Germany; 19 Department of Gynecology and Obstetrics, Helios Clinics Berlin-Buch, Berlin, Germany; 20 Department of Gynecology and Obstetrics, Ulm University Hospital, Ulm, Germany; 21 Department of Obstetrics and Gynecology, University Medical Center Freiburg, Freiburg, Germany; 22 Department of Gynecology and Obstetrics, Frauenklinik St. LouiseSt. Josefs-KrankenhausVincenz Kliniken Salzkotten + Paderborn, Paderborn, Germany; 23 Department of Gynecology and Obstetrics, Düsseldorf University Hospital, Düsseldorf, Germany; 24 Center for Integrated Oncology Aachen Bonn Köln Düsseldorf, Düsseldorf, Germany

**Keywords:** Metastatic breast cancer, Physical activity, Molecular subtype, IPAQ

## Abstract

**Background:**

Although adequate physical activity has been shown to be beneficial in early breast cancer, evidence in metastatic breast cancer is sparse and contradictory, which could be related to distinct effects of physical activity on the different molecular cancer subtypes. Therefore, we here evaluated the effect of physical activity on progression-free and overall survival (PFS, OS) in metastatic breast cancer, specifically looking at molecular subtypes.

**Methods:**

International Physical Activity Questionnaire (IPAQ) questionnaires, filled out by patients enrolled in the prospective PRAEGNANT registry (NCT02338167; n = 1,270) were used to calculate metabolic equivalent task (MET) minutes, which were subsequently categorized into low (n = 138), moderate (n = 995) or high IPAQ categories (n = 137). Cox regression analyses were used to evaluate the impact of IPAQ categories and its interaction with molecular subtypes on PFS and OS.

**Results:**

Patient and tumor characteristics were equally distributed across IPAQ categories. HER2pos, HRpos and TNBC were present in 23.1%, 65.7% and 11.2% of patients, respectively. IPAQ scores did not have an impact on PFS and OS in addition to established prognostic factors, either overall or in particular molecular subtypes (PFS: p = 0.33 and OS: p = 0.08, likelihood ratio test). Exploratory analyses showed higher overall survival rates for high IPAQ categories compared to low/moderate IPAQ categories in luminal B-like breast cancer.

**Conclusions:**

Self-reported physical activity using the IPAQ questionnaire did not significantly affect PFS or OS in patients suffering from metastatic breast cancer. Nevertheless, some hypothesis-generating differences between molecular subtypes could be observed, which may be interesting to evaluate further.

**Supplementary Information:**

The online version contains supplementary material available at 10.1186/s12885-024-13038-3.

## Background

Breast cancer is the most common cancer in women. In Germany, 72,000 patients are newly diagnosed with breast cancer every year, with around 14,000 cases being attributed to advanced or metastatic breast cancer [1]. Despite therapeutic advances, the average overall survival (OS) of patients suffering from metastatic breast cancer still lies around 3–4 years [2]. In an attempt to further improve prognosis independently from the development of new and advanced pharmaceutical therapies, research has focused on the identification of modifiable lifestyle factors, that could influence overall prognosis.

In recent years, physical activity has been identified as a one such modifiable lifestyle factor in breast cancer patients. Various studies have reported benefits with adequate activity and exercise. Compared to less active patients, active breast cancer patients have been shown to report improvements in quality of life and psychological wellbeing [3–6]. Furthermore, positive effects of physical activity on survival and disease recurrence have also been described [7, 8]. Most studies have focused on elucidating the role of physical activity in early breast cancer, while studies in advanced or metastatic breast cancer are still sparse. In addition, the exact effect of physical activity on survival and disease recurrence in the metastatic setting remains unclear, as contrasting results have been reported [9–11].

The different molecular subtypes of breast cancer (HER2-positive (HER2pos), hormone receptor-positive (HRpos; containing Luminal A-like and Luminal B-like breast cancer) and triple negative breast cancer (TNBC)) exhibit profound intrinsic differences. Not only are the underlying pathways that lead to pathology distinct, overall survival rates also vary across molecular cancer subtypes [12]. Here, Luminal A-like HRpos breast cancer has the best prognosis and TNBC the poorest prognosis [12, 13]. Notably, physical activity affects an array of metabolic pathways. For example, physical activity and exercise can indirectly decrease the level of circulating sex hormones by reducing visceral fat mass and and limiting adipocyte estrogen synthesis, while also stimulating insulin sensitivity and affecting the immune system [14, 15]. Estrogen signaling, insulin signaling and the immune system are indeed to a greater or lesser extent involved in the different cancer subtypes [12]. Hence, discrepancies in study results could in part be related to the diverse effects of physical activity on the different cancer subtypes. Thus far, subgroup-analyses have also yielded contradictory results. One study in metastatic breast cancer reported improved survival only in HER2pos breast cancer patients, whereas a meta-analysis showed a survival benefit in active patients with estrogen receptor (ER)-positive breast cancer, but not in ER-negative breast cancer [9, 16]. As such, there is a need for additional studies.

Using the prospective PRAEGNANT registry, we here evaluated the effect of physical activity, as self-reported by patients with the validated International Physical Activity Questionnaire (IPAQ), on progression-free survival (PFS) and OS in metastatic breast cancer and its interaction with the different molecular subtypes of breast cancer.

## Methods

### PRAEGNANT registry

The PRAEGNANT (Prospective Academic Translational Research Network for the Optimization of the Oncological Health Care Quality in the Adjuvant and Advanced/Metastatic Setting) observational patient registry (NCT02338167 [17]) focusses on prospectively collecting real-world data. The inclusion criteria for enrollment into the PRAEGNANT registry are as follows: 18 years or older, diagnosis of invasive breast cancer (irrespective of breast cancer status e.g. TNM, receptor status etc.), willingness to sign informed consent form, metastatic or locally advanced disease proven by clinical measures (i.e. standard imaging). Exclusion criteria are as follow: unwillingness to sign informed consent form, non-eligibility for observation due to severe comorbidities or unavailability according to the treating physician. Recruitment started in July 2014. All enrolled patients provided written informed consent and the study was approved by the Ethics Committee of the Medical Faculty, University of Tübingen, Tübingen, Germany (ethical approval number: 234/2014BO1: first approval on June 17 2014, approval of Amendment 1 on June 11 2015, approval of Amendment 2 on March 18 2019; Ethics Committee of the Medical Faculty, University of Tübingen, Tübingen, Germany) and all relevant ethics committees of participating sites.

#### Patient selection

**Fig. 1:**
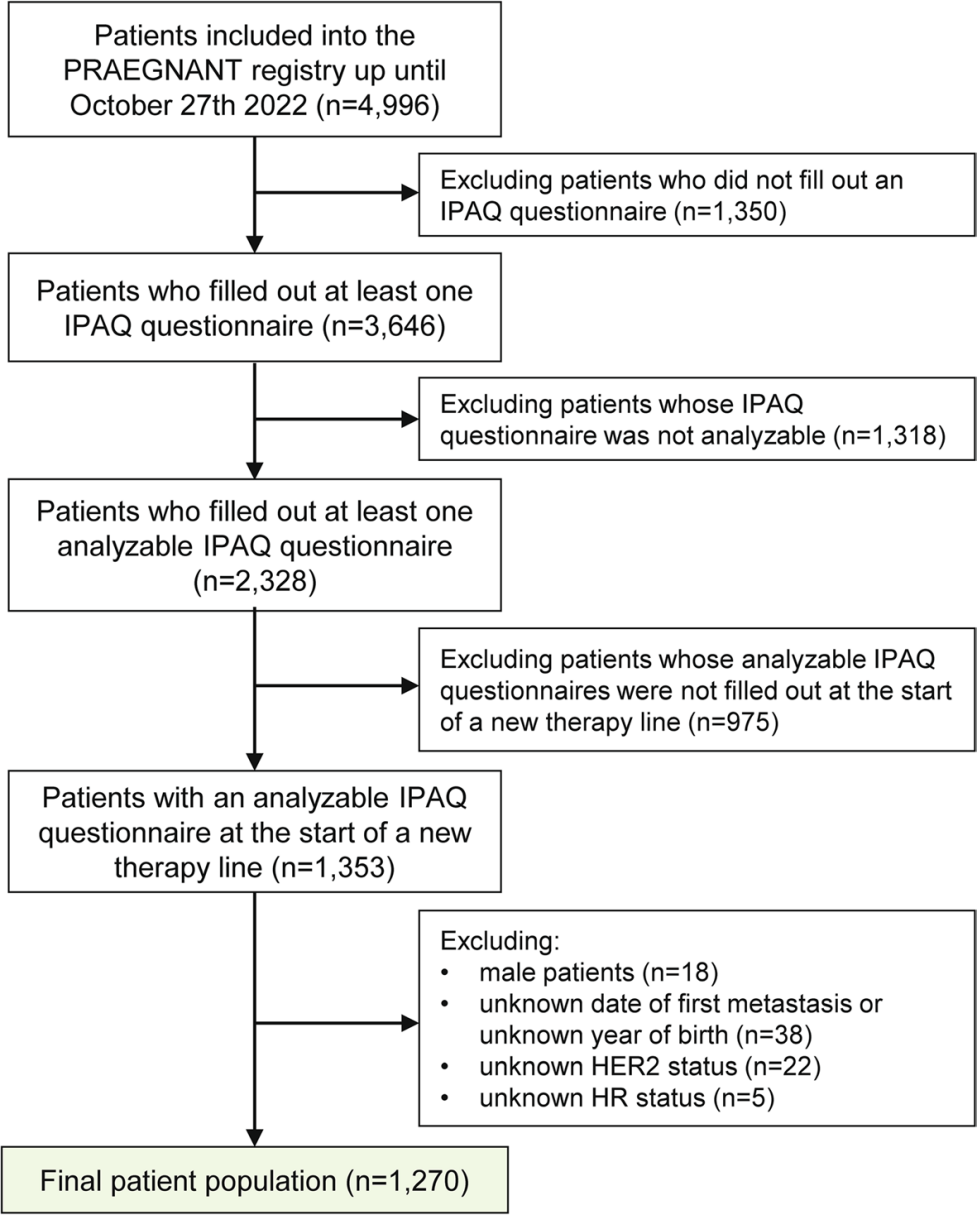
Patient flow chart. [IPAQ: International Physical Activity Questionnaire; HER2: human epidermal growth factor receptor 2, HR: hormone receptor]

Between July 2014 and October 2022, 4,996 patients from 61 study sites were included into the PRAEGNANT registry. Patient selection for this retrospective analysis of prospectively collected data in the PRAEGNANT registry was based on the completion of an analyzable IPAQ questionnaire at the start of a new therapy line (-90 days to + 30 days). Patients were excluded in the following order: 1,350 patients who did not fill out any IPAQ questionnaire, 1,318 patients who did not have an analyzable IPAQ questionnaire, 975 patients whose analyzable questionnaire was not filled out at the start of a new therapy line, 18 male patients, 38 patients from whom the first date of metastasis was unknown, 22 patients with unknown HER2 status and 5 patients with unknown hormone receptor (HR) status. A flow chart is presented in Fig. 1.

### Data collection

Data collected as part of the clinical routine (patient characteristics, current and previous disease status, imaging for disease staging and reevaluation, histology, current and previous therapies, etc.) was documented in an electronic case report form by trained staff. Automated plausibility checks (regarding completeness and consistence of case report form inputs) and on-site monitoring were performed. Data not commonly documented as part of routine clinical work was collected prospectively using structured questionnaires completed on paper (epidemiological data such as family history, cancer risk factors, quality of life, nutrition and lifestyle items, and psychological health). In the PRAEGNANT registry study, the short form of the IPAQ questionnaire was used to determine the patients’ habitual physical activity level [18]. This self-administered questionnaire contains questions on the physical activity during the last seven days and assesses the frequency and duration of sitting, walking, moderate-intensity activities and vigorous-intensity activities. IPAQ questionnaires were provided to patients at inclusion into the registry and at the time of disease progression. Supplemental Table 1 provides an overview of the data collected.

### Data preparation for analyses

#### HR status, HER2 Status, and Grading

The definition of HR status, HER2 status, and grading has been described previously [19]. In short, if an immunohistological biomarker assessment of the metastatic site was available, this receptor status was used for the analysis. If not, patients who received endocrine therapy in the metastatic setting were considered to be HRpos, and patients who had ever received anti-HER2 therapy were considered to be HER2pos. In case no information on histology or therapy for metastases was available, the most recent biomarker result from the primary tumor was used. There was no central review of biomarkers. The study protocol recommended assessing estrogen receptor and progesterone receptor status as positive if ≥ 1% was stained. A positive HER2 status required an immunohistochemistry score of 3 + or positive fluorescence in situ hybridization/chromogenic in situ hybridization (FISH/CISH). Based on HR status, HER2 status and grading, the categorial variable *molecular subtype* with the values “HER2pos”, “HRpos: Luminal A-like” (HRpos, HER2neg, grading 1 or 2), “HRpos: Luminal B-like” (HRpos, HER2neg, grading 3) and “triple negative” (HRneg, HER2neg) was constructed.

#### IPAQ categories

Based on the patients´ responses, the metabolic equivalent task (MET) minutes, describing the amount of energy expenditure during one minute, were calculated. Physical activity was further categorized in low, moderate or high. Low indicated that the level of physical activity did not meet the criteria for the moderate or high categories. Physical activity was classified as moderate when there were ≥ 3 days of vigorous intensity activity and/or walking during ≥ 30 min per day, or ≥ 5 days of moderate intensity activity and/or walking during ≥ 30 min per day, or ≥ 5 days of any combination of walking, moderate intensity or vigorous intensity activities with a total of ≥ 600 MET minutes a week. The high category comprised of either vigorous intensity activity on ≥ 3 days at a total of ≥ 1500 MET minutes a week, or ≥ 7 days of any combination of walking, moderate-intensity or vigorous-intensity activities at a total of ≥ 3000 MET minutes a week. If a patient had completed multiple IPAQ questionnaires during the course of their illness, which were analyzable and were filled-in at the start of a new therapy, the first questionnaire at the earliest therapy line start was used in the analyses.

#### Additional characteristics

The continuous variables *age at study entry* and *body mass index* (BMI) where captured within the study. Furthermore, the categorial variables *metastasis group* (bone only, brain, no brain and visceral but other, no brain but visceral), and *therapy* (anti-HER2 therapy, anti-hormone therapy, bevacizumab, CDK4/6-inhibitor, chemotherapy, mTOR-inhibitor, other) where surveyed. Finally, the ordinal variables *ECOG* status (0, 1, 2, 3, 4) and *therapy line* (1st, 2nd, 3rd, 4th or more) were collected.

#### Outcome parameter

PFS was defined from the date of therapy begin to the earliest date of disease progression or the last date known to be progression-free. For determining the earlies date of disease progression, all occurrences of distant-metastases, local recurrence and death from any cause were taken into account. PFS was censored at 5 years, and it was left-truncated for time to enter the study if the entry was after therapy begin. OS was defined from the date of therapy begin to the date of death from any cause or the last date known to be alive. OS was censored at 5 years, and it was left-truncated for time to enter the study if the entry was after therapy begin.

### Statistical analysis

The primary objective was to investigate whether IPAQ has an impact on PFS in the total patient population and particularly in molecular subtypes, in addition to other well-known prognostic factors. For this purpose, a multiple Cox regression model was fitted with PFS as the outcome and the following predictors: age at study entry, body mass index (BMI), molecular subtype, therapy line and metastasis group (*the basic model*). The type of received anti-cancer therapy was not considered as predictor in the basic model due to strong correlations with the molecular subtype (Supplemental Table 2). A second Cox regression model was fitted containing the predictor IPAQ and the interaction between IPAQ and molecular subtype, in addition to the predictors of the basic model (*the full model*). Both models were compared using the likelihood ratio test (LRT). A significant test result would indicate that IPAQ influenced PFS, either across all patients or within at least one molecular subtype. In case of significance, molecular subtype-specific adjusted hazard ratios for IPAQ (moderate vs, low, high vs. low) were estimated, using the full model. In case the LRT was not significant, no further statistical tests were performed to avoid false-positive results. Adjusted overall hazard ratios for IPAQ were estimated, using a reduced full model in which the interaction term was excluded (i.e., basic model plus IPAQ). The proportional hazards assumptions were checked using the Grambsch-Therneau method [20]. Missing values for predictors other than IPAQ, survival data or molecular subtype were imputed as done previously [21].

A similar analysis was performed for OS. As sensitivity analysis, corresponding unadjusted hazard ratios were estimated for PFS and OS, using a Cox regression model with IPAQ and, where appropriate, the interaction between IPAQ and molecular subtype. Unadjusted survival rates with 95% confidence intervals (CIs) and median survival times were estimated using the Kaplan–Meier product limit method. The 95% CI of median survival time was computed using the method of Brookmeyer and Crowley [22].

All of the tests were two-sided, and a *P*-value of < 0.05 was regarded as statistically significant. Calculations were carried out using the R-system for statistical computing (version 4.3.0; R Development Core Team, Vienna, Austria, 2023).

## Results

### Patient characteristics

Patient characteristics of the overall population, as well as the patient subgroups based on IPAQ categories are presented in Table 1. The low IPAQ category comprised 138 patients (10.9%), the moderate category 995 patients (78.3%) and the high category 137 (10.8%) patients. Age, BMI, molecular tumor subtype, therapy line, metastasis group, and type of anti-cancer therapy were equally distributed between the IPAQ categories. In the overall population, patients were 58.7 years (standard deviation (SD) 12.4 years) old and had a BMI of 25.9 kg/m^2^ (SD 5.3 kg/m^2^). The HRpos tumor subtype was present in 793 (66%) patients, whereas 278 (23%) tumors were HER2pos and 135 (11%) tumors were triple-negative. The majority of patients (37%) received first-line therapy at the time of IPAQ completion, whereas 25%, 15% and 23% of IPAQ questionnaires were respectively filled out at the start of second-line, third-line or higher-line therapy. The most common type or metastasis were visceral metastasis, which were present 53.7% of all patients. Bone metastasis were present in 18.9% of patients and brain metastasis in 9.3% of patients. 34.6% of patients received chemotherapy, whereas anti-HER2 therapy (17% of patients), anti-hormone therapy (15% of patients) and CDK4/6-inhibtor therapy (18% of patients) were the other most common anti-cancer therapies. The large majority of patients (91.8%) had no or minor physical limitations (ECOG 0 in 49.3% of patients and ECOG 1 in 42.5% of patients). In contrast to all other patient characteristics, some imbalances in ECOG status were observed across IPAQ categories. The low IPAQ category had the largest percentage of patients with a higher ECOG status (ECOG 2–4 IPAQ low category: 22% of patients; IPAQ moderate category: 7% of patients; IPAQ high category: 4% of patients), while ECOG status was comparable in the moderate and high IPAQ categories (Table 1). As expected, the type of anti-cancer therapy corresponded to the cancer molecular subtype (Supplemental Table 1).

**Table 1:** Patient characteristics, showing mean and standard deviation or frequency and percentage

	IPAQ category			All (*n* = 1,270)
	low (*n* = 138)	moderate (*n* = 995)	high (*n* = 137)	
Age at study entry (years)—mean (SD)	60.5 (12.6)	58.6 (12.4)	57.7 (11.9)	58.7 (12.4)
BMI (kg/m^2^)—mean (SD)	25.6 (5.5)	25.9 (5.4)	25.6 (4.7)	25.9 (5.3)
Molecular tumor subtype—n (%)				
HER2pos	26 (20.0)	217 (23.1)	35 (25.7)	278 (23.1)
HRpos: Luminal A-like	75 (57.7)	425 (45.2)	49 (36.0)	549 (45.5)
HRpos: Luminal B-like	18 (13.8)	194 (20.6)	32 (23.5)	244 (20.2)
TNBC	11 (8.5)	104 (11.1)	20 (14.7)	135 (11.2)
Missing	8	55	1	64
Therapy line – n (%)				
1	41 (29.7)	394 (39.6)	40 (29.2)	475 (37.4)
2	39 (28.3)	241 (24.2)	35 (25.5)	315 (24.8)
3	19 (13.8)	144 (14.5)	22 (16.1)	185 (14.6)
4 +	39 (28.3)	216 (21.7)	40 (29.2)	295 (23.2)
Metastasis pattern				
Brain	12 (8.8)	93 (9.5)	11 (8.1)	116 (9.3)
Bone	27 (19.7)	187 (19.1)	23 (17.0)	237 (18.9)
Visceral	76 (55.5)	523 (53.3)	74 (54.8)	673 (53.7)
Other	22 (16.1)	178 (18.1)	27 (20.0)	227 (18.1)
Missing	1	14	2	17
ECOG status				
0	32 (32.0)	379 (50.7)	57 (55.9)	468 (49.3)
1	47 (47.0)	316 (42.2)	41 (40.2)	404 (42.5)
2	18 (18.0)	50 (6.7)	3 (2.9)	71 (7.5)
3	2 (2.0)	3 (0.4)	1 (1.0)	6 (0.6)
4	1 (1.0)	0 (0)	0 (0)	1 (0.1)
Missing	38	247	35	320
Therapy				
Anti-HER2 therapy	17 (12.3)	180 (18.1)	25 (18.2)	222 (17.5)
Anti-hormone therapy	15 (10.9)	154 (15.5)	18 (13.1)	187 (14.7)
Bevacizumab	11 (8.0)	86 (8.6)	12 (8.8)	109 (8.6)
CDK4/6-inhibitor	30 (21.7)	174 (17.5)	22 (16.1)	226 (17.8)
Chemotherapy	52 (37.7)	337 (33.9)	50 (36.5)	439 (34.6)
mTOR-inhibitor	7 (5.1)	48 (4.8)	9 (6.6)	64 (5.0)
Other	6 (4.3)	16 (1.6)	1 (0.7)	23 (1.8)

*BMI* Body mass index, *IPAQ* International Physical Activity Questionnaire, *HER2* Human epidermal growth factor receptor 2, *TNBC* Triple negative breast cancer, *ECOG* Eastern Cooperative Oncology Group (ECOG) performance status, *SD* Standard deviation

### IPAQ-reported physical activity and progression-free survival

The median observation/follow-up time for PFS was 7.4 months (interquartile range (IQR) 3.4–16.9 months). An impact of IPAQ on PFS could not be shown, neither across all patients nor within molecular subtypes (p = 0.37, LRT). The adjusted hazard ratios were 1.07 (95% CI 0.88–1.32) when comparing the moderate versus low IPAQ categories and 1.11 (95% CI 0.85–1.44) between the high and low IPAQ categories. Unadjusted hazard ratios showed similar results and are presented in Table 2.

**Table 2:** Cox regression analyses, showing adjusted and unadjusted hazard ratios for IPAQ categories

Outcome	IPAQ category	Adjusted^a^ hazard ratios (95% CI)	Unadjusted hazard ratios (95% CI)
PFS	moderate vs. low	1.07 (0.88, 1.32)	1.02 (0.84, 1.24)
	high vs. low	1.11 (0.85, 1.44)	1.18 (0.92, 1.52)
OS	moderate vs. low	0.95 (0.75, 1.21)	0.93 (0.74, 1.16)
	high vs. low	0.71 (0.51, 0.97)	0.79 (0.58, 1.08)

*PFS* Progression free survival, *OS* Overall survival, *IPAQ* International Physical Activity Questionnaire, *CI* Confidence interval

^a^Hazard ratios were adjusted for age, body mass index, molecular subtype, therapy line and metastasis group. Since the likelihood ratio tests did not show a significant impact of IPAQ on PFS or OS, the confidence intervals should be interpreted with caution. That is, a confidence interval that does not include 1 does not imply that there is a significant difference in survival

**Fig. 2:**
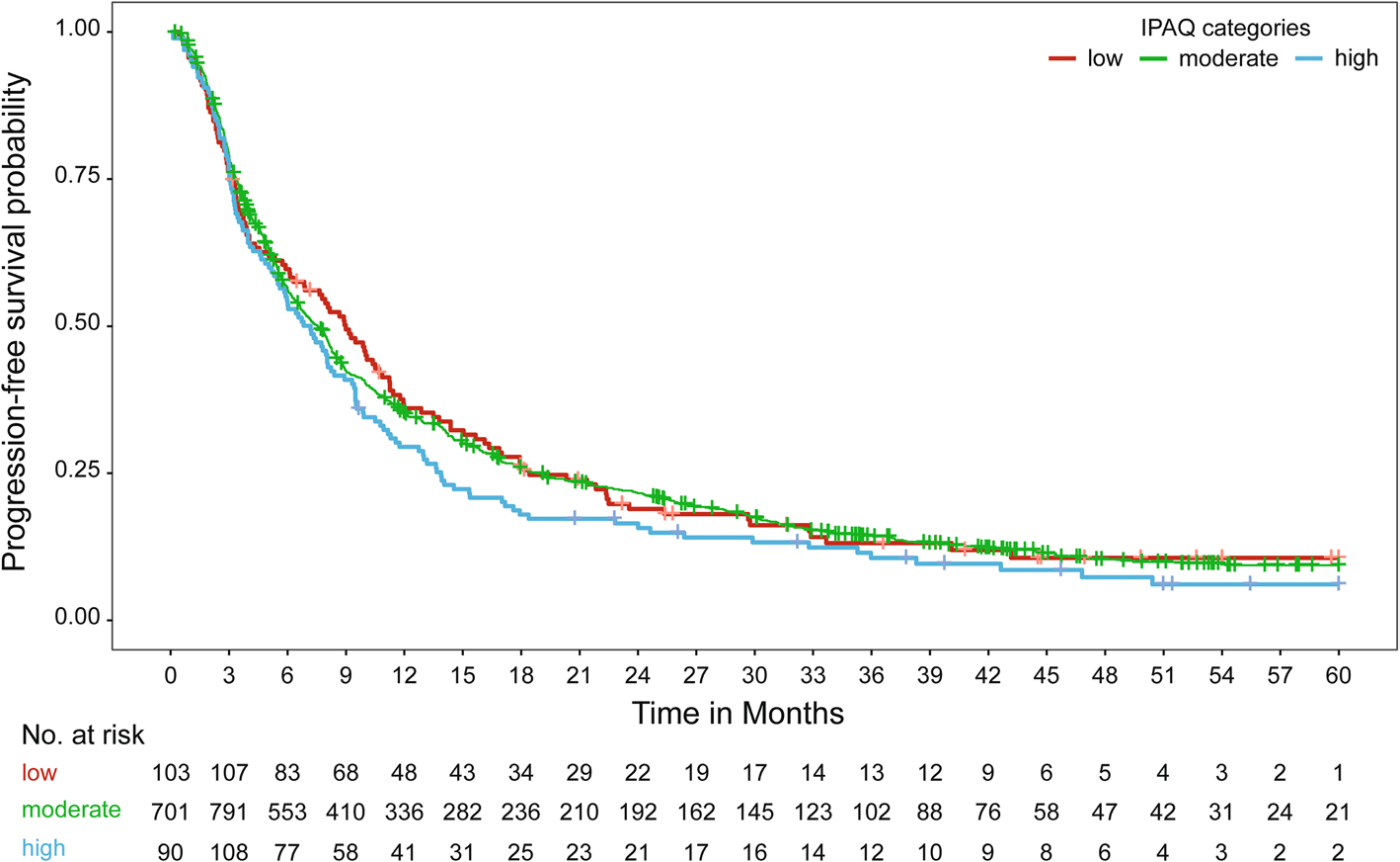
Kaplan–Meier curve for progression-free survival relative to IPAQ categories [IPAQ: International Physical Activity Questionnaire]

**Fig. 3:**
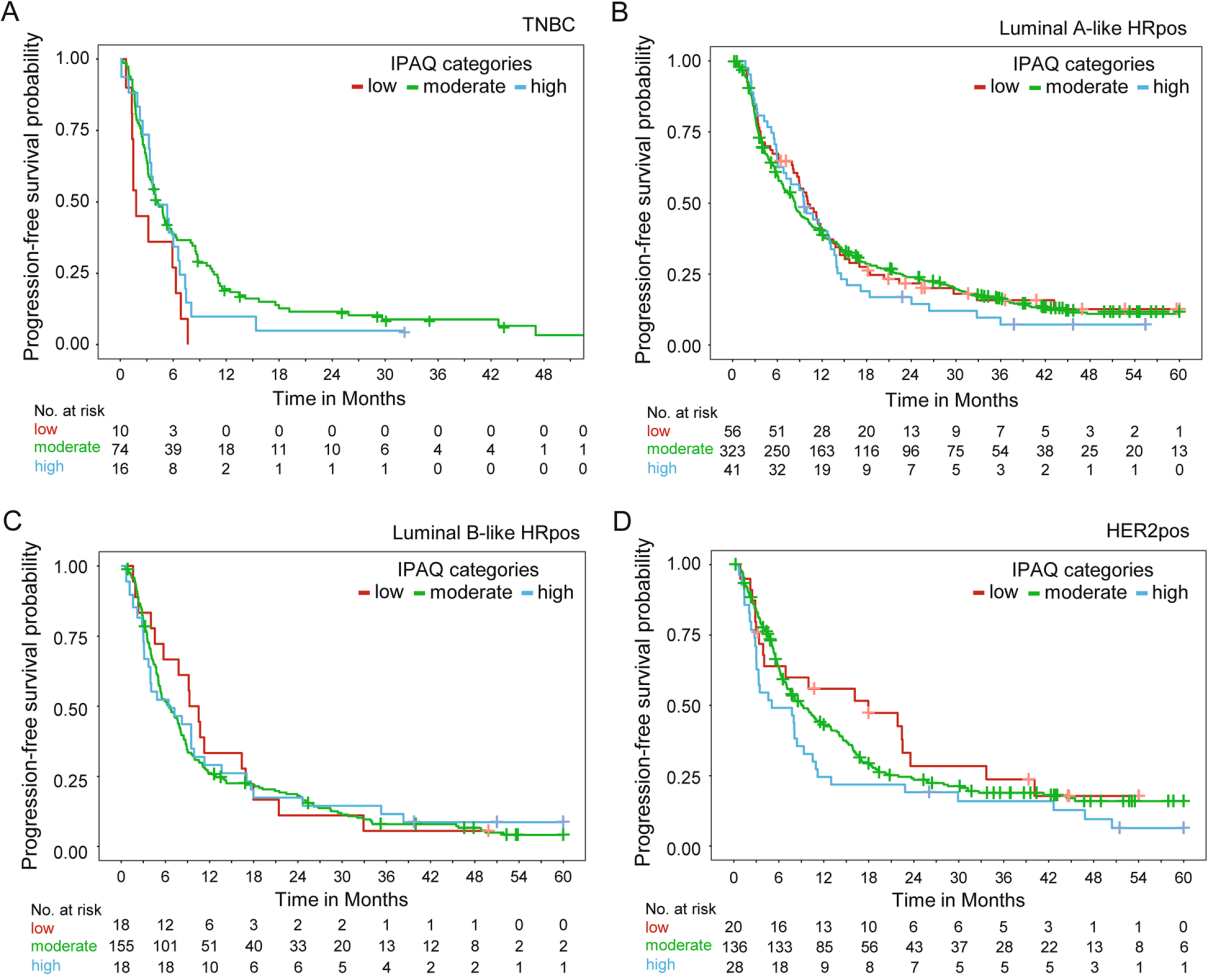
Kaplan–Meier curves for progression-free survival relative to IPAQ categories in patient subgroups based on molecular subtype. **A** Patients with triple negative breast cancer (TNBC). **B** Patients with hormone receptor-positive (HRpos) luminal A-like breast cancer. **C** Patients with HRpos luminal B-like breast cancer. **D** Patients with HER2-positive (HER2pos) breast cancer. [IPAQ: International Physical Activity Questionnaire]

Median survival times and survival rates relative to the IPAQ categories are presented in Table 3 and Fig. 2. Median survival times and survival rates relative to patient subgroups according to IPAQ categories and molecular subtypes are presented in Table 4 and Fig. 3.

**Table 3:** Median survival times and survival rates relative to IPAQ categories

Outcome	IPAQ	Patients	Events	Median survival time in months (95% CI)	6-month survival rate (95% CI)	12-month survival rate (95% CI)	24-month survival time (95% CI)	36-month survival rate (95% CI)
PFS	low	138	116	9.0 (6.3, 11.2)	0.60 (0.52, 0.69)	0.36 (0.29, 0.45)	0.19 (0.13, 0.27)	0.13 (0.08, 0.21)
	moderate	995	838	7.6 (6.7, 8.1)	0.57 (0.54, 0.60)	0.35 (0.32, 0.38)	0.22 (0.19, 0.24)	0.14 (0.12, 0.17)
	high	137	124	7.2 (5.5, 9.3)	0.54 (0.46, 0.63)	0.29 (0.23, 0.38)	0.16 (0.11, 0.24)	0.11 (0.06, 0.17)
OS	low	138	84	27.1 (20.4, 35.6)	0.89 (0.84, 0.95)	0.73 (0.65, 0.81)	0.52 (0.44, 0.62)	0.39 (0.31, 0.49)
	moderate	995	597	27.7 (24.6, 30.3)	0.90 (0.89, 0.92)	0.76 (0.73, 0.78)	0.54 (0.51, 0.57)	0.41 (0.38, 0.44)
	high	137	74	33.5 (25.7, 40.0)	0.93 (0.88, 0.97)	0.83 (0.76, 0.90)	0.62 (0.54, 0.71)	0.46 (0.38, 0.56)

*PFS* Progression free survival, *OS* Overall survival, *IPAQ* International Physical Activity Questionnaire, *CI* Confidence interval

**Table 4:** Median progression-free survival times and survival rates relative to molecular subtypes and IPAQ categories

Molecular subtype	IPAQ	Patients	Events	Median survival time (months) (95% CI)	6-month survival rate (95% CI)	12-month survival rate (95% CI)	24-month survival rate (95% CI)	36-month survival rate (95% CI)
HRpos: Luminal A-like	low	75	61	10.1 (8.7, 13.5)	0.67 (0.57, 0.79)	0.39 (0.29, 0.51)	0.22 (0.14, 0.34)	0.16 (0.09, 0.28)
	moderate	425	354	8.3 (7.2, 9.9)	0.60 (0.55, 0.65)	0.39 (0.35, 0.44)	0.25 (0.21, 0.29)	0.16 (0.13, 0.21)
	high	49	44	9.6 (6.8, 13.7)	0.65 (0.52, 0.80)	0.40 (0.28, 0.56)	0.17 (0.09, 0.32)	0.07 (0.02, 0.21)
HRpos: Luminal B-like	low	18	17	9.9 (5.7, 17.9)	0.67 (0.48, 0.92)	0.33 (0.17, 0.64)	0.11 (0.03, 0.41)	0.06 (0.01, 0.37)
	moderate	194	179	6.5 (5.3, 7.9)	0.52 (0.45, 0.60)	0.26 (0.21, 0.33)	0.18 (0.13, 0.25)	0.08 (0.05, 0.13)
	high	32	29	6.4 (3.7, 11.3)	0.52 (0.37, 0.74)	0.29 (0.17, 0.50)	0.17 (0.08, 0.36)	0.12 (0.05, 0.29)
HER2pos	low	26	19	17.9 (4.0, 33.7)	0.64 (0.48, 0.86)	0.56 (0.39, 0.79)	0.28 (0.15, 0.55)	0.24 (0.11, 0.50)
	moderate	217	166	9.0 (7.1, 12.2)	0.63 (0.57, 0.70)	0.43 (0.37, 0.50)	0.23 (0.18, 0.30)	0.19 (0.14, 0.25)
	high	35	32	5.1 (3.0, 10.5)	0.49 (0.35, 0.69)	0.25 (0.14, 0.44)	0.19 (0.10, 0.37)	0.16 (0.07, 0.34)
TNBC	low	11	11	1.8 (1.3, NA)	0.27 (0.10, 0.71)	NA	NA	NA
	moderate	104	93	4.3 (3.2, 5.9)	0.39 (0.30, 0.49)	NA	NA	NA
	high	20	19	4.0 (3.4, 7.4)	0.39 (0.23, 0.68)	NA	NA	NA

*HER2pos* Human epidermal growth factor receptor 2 positive, *HRpos* Hormone receptor-positive, *TNBC* Triple negative breast cancer, *IPAQ* International Physical Activity Questionnaire, *CI* Confidence interval, *NA* Not applicable – could not be calculated

### IPAQ-reported physical activity and overall survival

**Fig. 4:**
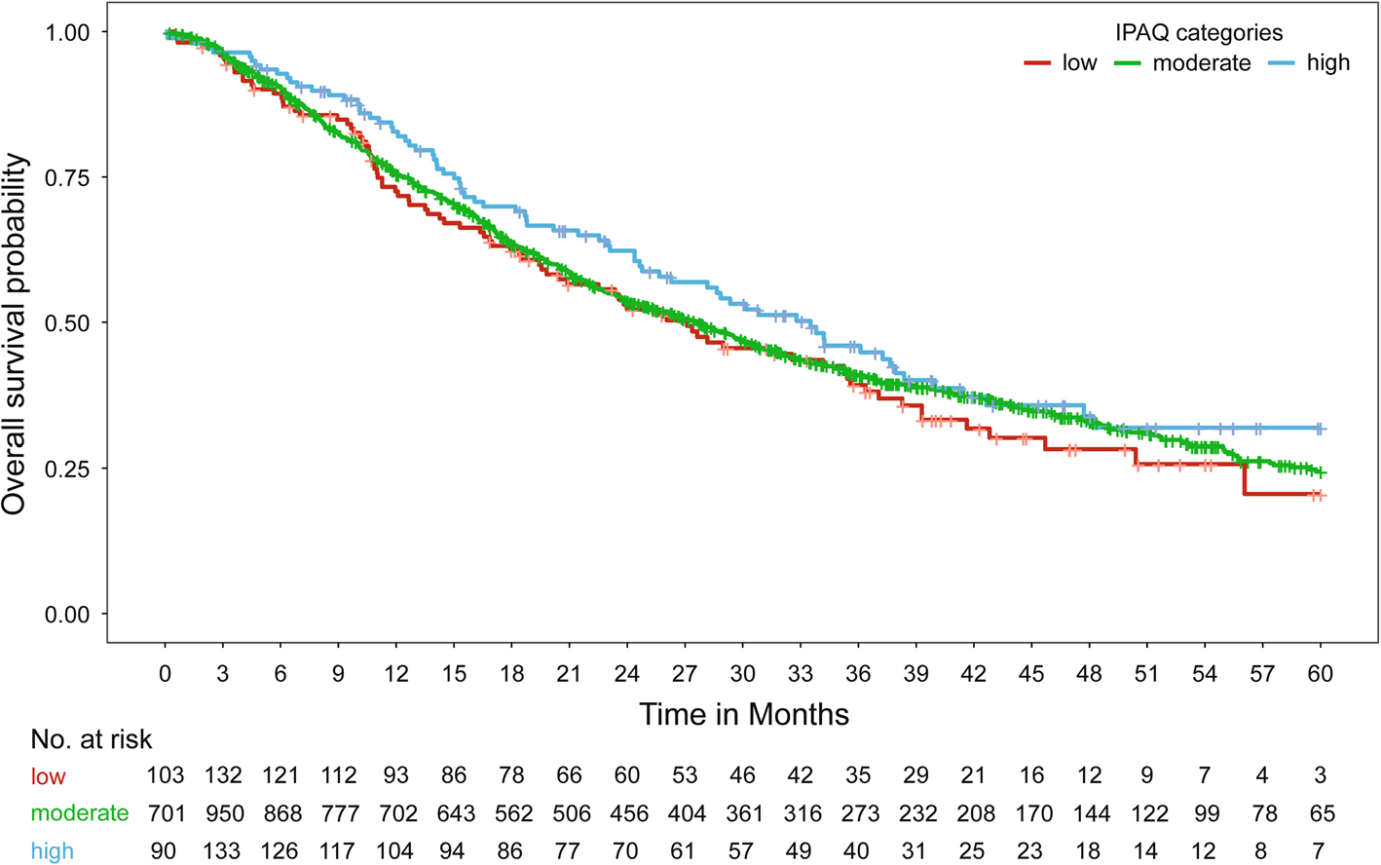
Kaplan–Meier curves for overall survival relative to IPAQ categories [IPAQ: International Physical Activity Questionnaire]

**Fig. 5:**
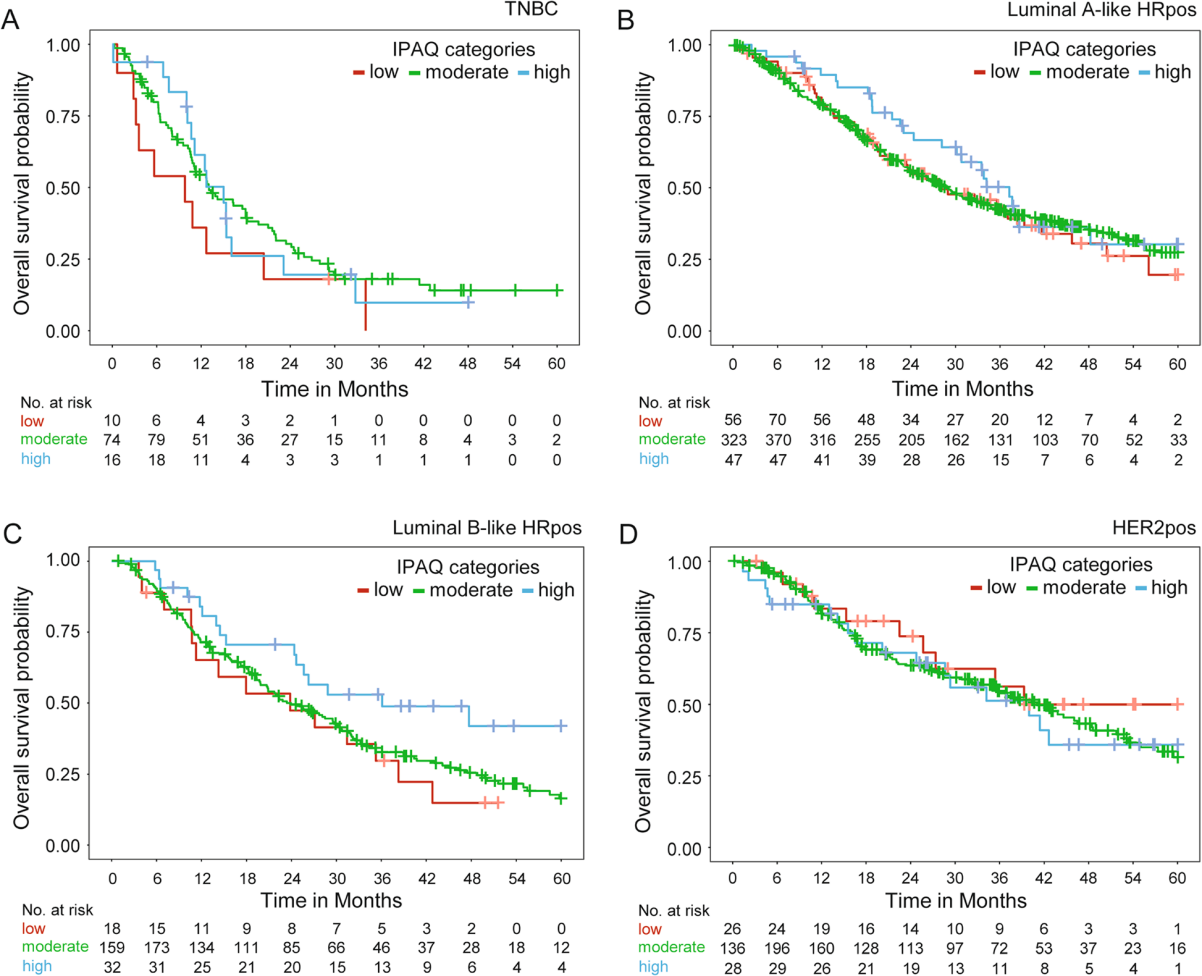
Kaplan–Meier curves for overall survival relative to IPAQ categories in patient subgroups based on molecular subtype. **A** Patients with triple negative breast cancer (TNBC). **B** Patients with hormone receptor-positive (HRpos) luminal A-like breast cancer. **C** Patients with HRpos luminal B-like breast cancer. **D** Patients with HER2-positive (HER2pos) breast cancer. [IPAQ: International Physical Activity Questionnaire]

Median observation/follow-up time for OS was 21.6 months (IQR 10.6–37.6 months). An impact of IPAQ on OS could also not be shown, neither across all patients nor within molecular subtypes (p = 0.08, LRT). The adjusted hazard ratios were 0.95 (95% CI 0.75–1.21) between the moderate and low IPAQ categories and 0.71 (95% CI 0.51–0.97) between the high and low IPAQ categories. Unadjusted hazard ratios are presented in Table 2. Median OS time and OS rates did not vary greatly between the IPAQ categories, although the highest survival rates were consistently observed in the high IPAQ category (Table 3 and Fig. 4). Only in patients with luminal B-like breast cancer, a higher survival rate in the high IPAQ category compared to the low/moderate IPAQ categories was observed (Table 5 and Fig. 5).

**Table 5:** Median overall survival times and survival rates relative to molecular subtypes and IPAQ categories

Molecular subtype	IPAQ	Patients	Events	Median survival time in months (95% CI)	6-month survival rate (95% CI)	12-month survival rate (95% CI)	24-month survival rate (95% CI)	36-month survival rate (95% CI)
HRpos: Luminal A-like	low	75	44	28.2 (20.9, 41.6)	0.94 (0.89, 1.00)	0.80 (0.71, 0.90)	0.55 (0.44, 0.68)	0.44 (0.33, 0.58)
	moderate	425	251	28.6 (25.1, 33.5)	0.90 (0.88, 0.93)	0.79 (0.75, 0.83)	0.56 (0.51, 0.61)	0.42 (0.38, 0.48)
	high	49	26	37.3 (30.2, NA)	0.96 (0.90, 1.00)	0.89 (0.81, 0.99)	0.69 (0.57, 0.84)	0.50 (0.37, 0.68)
HRpos: Luminal B-like	low	18	14	23.8 (11.3, NA)	0.89 (0.75, 1.00)	0.65 (0.46, 0.92)	0.47 (0.29, 0.78)	0.30 (0.14, 0.62)
	moderate	194	136	24.0 (19.9, 30.0)	0.90 (0.86, 0.95)	0.71 (0.65, 0.78)	0.49 (0.43, 0.57)	0.33 (0.26, 0.41)
	high	32	16	36.1 (24.6, NA)	0.97 (0.91, 1.00)	0.84 (0.72, 0.98)	0.71 (0.56, 0.89)	0.53 (0.38, 0.74)
HER2pos	low	26	10	39.3 (27.4, NA)	0.96 (0.89, 1.00)	0.83 (0.70, 1.00)	0.74 (0.58, 0.94)	0.56 (0.38, 0.84)
	moderate	217	105	41.0 (35.4, 51.7)	0.95 (0.92, 0.98)	0.82 (0.76, 0.87)	0.64 (0.57, 0.71)	0.54 (0.47, 0.62)
	high	35	17	40.0 (24.8, NA)	0.85 (0.74, 0.98)	0.85 (0.74, 0.98)	0.68 (0.53, 0.87)	0.51 (0.35, 0.74)
TNBC	low	11	10	9.8 (3.2, NA)	0.54 (0.31, 0.93)	0.36 (0.16, 0.79)	0.18 (0.05, 0.63)	NA
	moderate	104	79	13.0 (10.8, 18.2)	0.80 (0.72, 0.88)	0.54 (0.45, 0.65)	0.30 (0.22, 0.41)	NA
	high	20	15	15.0 (10.6, NA)	0.94 (0.83, 1.00)	0.61 (0.43, 0.88)	0.20 (0.07, 0.52)	NA

*HER2pos* Human epidermal growth factor receptor 2 positive, *HRpos* Hormone receptor-positive, *TNBC* Triple negative breast cancer, *IPAQ* International Physical Activity Questionnaire, *CI* Confidence interval, *NA* Not applicable – could not be calculated

## Discussion

In this study, we evaluated the effect of physical activity, as self-reported with the IPAQ questionnaire, on PFS and OS in metastatic breast cancer. We could not show that IPAQ scores had an impact on progression-free and overall survival in addition to established prognostic factors, either overall or in particular molecular subtypes. However, exploratory analyses suggest that it may be interesting to further evaluate the effect of physical activity on outcome in patients with luminal B-like breast cancer.

Abundant evidence links adequate physical activity to improved survival in early breast cancer [7, 8]. Patients with metastatic breast cancer have often been excluded from studies on physical activity as the comorbidities associated with metastatic disease, the treatment-related side effects and the location of the metastasis, could contribute to an altered, and often already reduced, physical activity level. To date, results of the few studies evaluating the effect of physical activity on both PFS and OS in metastatic breast cancer have been contradictory. Whereas clear beneficial effects are reported by some studies, others could not observe any benefit [9–11, 23]. Additionally, studies considering the different molecular subtypes of metastatic breast cancer are even sparser. A recent study showed improved OS with high physical activity irrespective of molecular subtype with an unadjusted model. After adjustment, only patients with HER2pos breast cancer showed a survival benefit [9]. In our adjusted prediction model, IPAQ categories did not significantly impact PFS and OS, neither across all patients, nor within molecular subtypes. The potential bias from the received anti-cancer therapy has to be noted. The regression model did not contain the type of anti-cancer therapy the patient received, as this is highly dependent on the molecular subtype of the cancer. Although new and efficient therapies that affect both PFS and OS have recently been introduced for all breast cancer patients, the relative prognostic benefit of new therapies varied across molecular subtypes. In HER2pos metastatic disease, the introduction of pertuzumab and trastuzumab in combination to chemotherapy improved median survival to 56.5 months, whereas earlier estimated survival was around 2 years [24]. Even more advanced therapies, such as trastuzumab-emtansine and trastuzumab-deruxtecan have also shown profound improvements in both PFS and OS [25, 26]. In comparison, in patients with HRpos disease, an overall improvement in OS of 7 months was observed between patients diagnosed in 2008–2010 and 2017–2019 [27]. As patients were included into our registry from 2014 onwards, the heterogeneity in received medications could have influenced prognosis. Furthermore, some imbalances in performance status (ECOG) were observed across IPAQ categories. Compared to the intermediate and high IPAQ categories, the low IPAQ category consisted of more patients with a higher ECOG status. The intermediate and high IPAQ categories were however comparable. As the level of physical activity is at least in part dependent on the performance status, this finding is not unexpected. It has to be noted that majority of patients across IPAQ categories had ECOG status 0–1. Nevertheless, bias could have been introduced as ECOG is also associated with prognosis. Although we included known and available predictors as adjustment variables in our prediction model, it is possible that other factors influencing physical activity and prognosis may exist. However, minor influential confounders could have reduced the statistical power, which is why we did not consider additional factors.

Exploratory analyses hinted towards an impact of physical activity on outcome in luminal B-like breast cancer, which could serve hypothesis-generating. Nevertheless, considering the absence of a clear prognostic effect of physical activity in this study, it is possible that the benefit of physical activity for patients with metastatic breast cancer lies in affecting parameter other than PFS and OS, such as quality of life or fatigue. Reduced fatigue and improved quality of life with exercise has indeed been observed in patients suffering from metastatic breast cancer [28, 29].

Some additional limitations have to be addressed. First, despite its wide-spread use, questionnaire-reported physical activity has its limitations. Overestimation of physical activity is a known problem that has been noted in the IPAQ short and long form, as well as in breast cancer patients [30–33]. Another problem is the high number of non-analyzable questionnaires. In this study, 36% of patients that filled out an IPAQ questionnaire had to be excluded from analysis due to not having at least one analyzable questionnaire. Here, questionnaires could not be analyzed due to missing values, which could have introduced bias. Due to the small number of patients who filled out the IPAQ questionnaire upon inclusion and at disease progression, longitudinal evaluation of the level of physical activity was not possible. Last, whereas the use of the low, moderate and high categories is recommended and simplifies data interpretation, the corresponding skewedness of the data results in unequal group sizes, which could limit the statistical power in subgroup analyses. Furthermore, categorization also precludes discrimination of the effect of the intensity or the duration of physical activity, as a combination of both is used to establish IPAQ categories. To better evaluate the effect of physical activity on breast cancer survival, interventional studies using objective measurements of physical activity, potentially discriminating between the duration and intensity of physical activity, should be conducted.

## Conclusions

Self-reported physical activity using the IPAQ questionnaire did not significantly affect progression-free or overall survival in patients suffering from metastatic breast cancer. Nevertheless, exploratory analyses hinted to the potential benefit of future studies evaluating the effect of physical activity on prognosis in luminal B-like metastatic breast cancer.

## Supplementary Information

Supplementary Material 1.

## Data Availability

The datasets used and/or analyzed during the current study are available from the corresponding author on reasonable request.

## Abbreviations

BMI: Body mass index
CI: Confidence Interval
CISH: Chromogenic in situ hybridization
ER: Estrogen receptor
FISH: Fluorescence in situ hybridization
HER2pos: HER2-positive
HR: Hormone receptor
HRpos: Hormone receptor-positive
IPAQ: International Physical Activity Questionnaire
LRT: Likelihood ratio test
MET: Metabolic equivalent task
OS: Overall survival
PFS: Progression-free survival
SD: Standard deviation
