# Supplementary material for: The impact of physical activity on progression-free and overall survival in metastatic breast cancer based on molecular subtype
Source: BMC Cancer. 2024 Oct 16;24:1284. doi: 10.1186/s12885-024-13038-3 (PMC11481816; doi:10.1186/s12885-024-13038-3)
Supplement: Supplementary file 1 — Supplementary Material 1. [file 12885_2024_13038_MOESM1_ESM.docx]

**Supplementary Materials**

**The impact of physical activity on progression-free and overall survival in metastatic breast cancer based on molecular subtype**

Philipp Ziegler ^1^, Andreas D. Hartkopf ^2^, Markus Wallwiener ^3^, Lothar Häberle ^1,4^, Hans-Christian Kolberg ^5^, Peyman Hadji ^6^, Hans Tesch ^7^, Johannes Ettl ^8,9^, Diana Lüftner ^10^, Volkmar Müller ^11^, Laura L. Michel ^12^, Erik Belleville ^13^, Pauline Wimberger ^14^, Carsten Hielscher ^15^, Hanna Huebner ^1^, Sabrina Uhrig ^1^, Lena A. Wurmthaler ^1^, Carolin C. Hack ^1^, Christoph Mundhenke ^16^, Christian Kurbacher ^76^, Peter A. Fasching ^1*^, Rachel Wuerstlein ^18^, Michael Untch ^19^, Wolfgang Janni ^20^, Florin-Andrei Taran ^21^, Michael P.  Lux ^22^, Diethelm Wallwiener ^2^, Sara Y. Brucker ^2^, Tanja N.  Fehm ^23,24^, Andreas Schneeweiss ^12^, Chloë Goossens ^1^

1 Department of Gynecology and Obstetrics, Erlangen University Hospital, Comprehensive Cancer Center Erlangen-EMN, Friedrich Alexander University of Erlangen–Nuremberg, Germany

2 Department of Obstetrics and Gynecology, University of Tübingen, Tübingen, Germany

3 Department of Gynecology, Halle University Hospital, Halle, Germany

4 Biostatistics Unit, Department of Gynecology and Obstetrics, Erlangen University Hospital, Erlangen, Germany

5 Department of Gynecology and Obstetrics, Marienhospital Bottrop, Bottrop, Germany

6 Frankfurt Center for Bone Health, Frankfurt am Main, Germany

7 Oncology Practice, Bethanien Hospital, Frankfurt am Main, Germany

8 Department of Obstetrics and Gynecology, Klinikum rechts der Isar, Technical University of Munich, Munich, Germany

9 Cancer Center Kempten/ Allgäu (CCKA), Klinikum Kempten, Kempten Germany

10 Immanuel Hospital Märkische Schweiz & Immanuel Campus Rüdersdorf, Medical University of Brandenburg Theodor-Fontane, Rüdersdorf bei Berlin, Germany

11 Department of Gynecology, Hamburg-Eppendorf University Medical Center, Hamburg, Germany

12 National Center for Tumor Diseases, Heidelberg University Hospital, German Cancer Research Center (DKFZ), Heidelberg, Germany

13 ClinSol GmbH & Co KG, Würzburg, Germany

14 National Center for Tumor Diseases Dresden and Department of Gynecology and Obstetrics, University Hospital Dresden, TU Dresden, Dresden, Germany

15 g.SUND Gynäkologie-Onkologisches Zentrum, Stralsund, Germany

16 Department of Gynecology and Obstetrics, Klinik Hohe Warte, Bayreuth, Germany

17 Department of Gynecology I (Gynecologic Oncology), Gynecologic Center Bonn-Friedensplatz, Bonn, Germany

18 Breast Center and CCC Munich, Dept of Gynecology and Obstetrics, University Hospital LMU Munich, Munich, Germany

19 Department of Gynecology and Obstetrics, Helios Clinics Berlin-Buch, Berlin, Germany

20 Department of Gynecology and Obstetrics, Ulm University Hospital, Ulm, Germany

21 Department of Obstetrics and Gynecology, University Medical Center Freiburg, Freiburg, Germany

22 Department of Gynecology and Obstetrics, Frauenklinik St. Louise, Paderborn, St. Josefs-Krankenhaus, Salzkotten, Germany; St. Vincenz Kliniken Salzkotten + Paderborn, Paderborn, Germany

23 Department of Gynecology and Obstetrics, Düsseldorf University Hospital, Düsseldorf, Germany

24 Center for integrated oncology Aachen Bonn Köln Düsseldorf, Düsseldorf, Germany

* corresponding author: Department of Gynecology and Obstetrics, Erlangen University Hospital; Comprehensive Cancer Center Erlangen EMN; Friedrich Alexander University of Erlangen–Nuremberg, Universitätsstraße 21–23, 91054 Erlangen, Germany. Tel: +49 (0)9131-85-33553 / fax: +49 (0)9131-85-33938. E-mail: peter.fasching@uk-erlangen.de

Supplemental table 1. **Data categories recorded in the PRAEGNANT study.**

| Data continuously captured, if applicable | Data assessed at study entry | Data assessed at follow-up care appointments |
| --- | --- | --- |
| Concomitant diseases | Life status, ECOG | Life status, ECOG |
| Concomitant medication | Quality of life | Quality of life |
| Cancer systemic therapies | Breast cancer risk factor questionnaire | Breast and axilla evaluation |
| Cancer radiotherapy | Breast and axilla evaluation | Distant metastasis evaluation |
| Cancer surgery | Distant metastasis evaluation | Biomaterial ascertainment |
| Breast cancer, right side | Biomaterial ascertainment | PRO questionnaires |
| Breast cancer, left side | PRO questionnaires |  |

ECOG, Eastern Cooperative Oncology Group (performance status); PRO, patient-reported outcome.

Supplemental table 2: **Type of anti-cancer therapy per molecular tumor subtype**

| Therapy type - n (%) | Molecular tumor subtype | | | | | |
| --- | --- | --- | --- | --- | --- | --- |
|  | HER2pos  (n=278) | HRpos:  Luminal A-like  (n=549) | HR pos: Luminal B-like  (n=244) | TNBC  (n=135) | Other  (n=64) |  |
| Anti-HER2 therapy | 66 (23.7) | 2 (0.4) | 1 (0.4) | 0 (0) | 0 |  |
| Anti-hormone therapy | 25 (9.0) | 304 (55.4) | 105 (43.0) | 7 (5.2) | 29 |  |
| Chemotherapy | 40 (14.4) | 191 (34.8) | 118 (48.4) | 114 (84.4) | 29 |  |
| Other | 147 (52.9) | 52 (9.5) | 20 (8.2) | 14 (10.4) | 6 |  |

[HER2pos: human epidermal growth factor receptor 2-positive, HRpos: hormone receptor-positive, TNBC: triple negative breast cancer]
